# Comparative effectiveness of different treatments for post-stroke insomnia: A network meta-analysis^☆^

**DOI:** 10.1016/j.heliyon.2023.e21801

**Published:** 2023-10-31

**Authors:** Zhaoming Song, Ying chen, Jian Li, Zhouqin Chen, Xiaojun Lu, Zhong Wang

**Affiliations:** a Department of Neurosurgery & Brain and Nerve Research Laboratory, The First Affiliated Hospital of Soochow University, Suzhou, Jiangsu Province, 215006, China; b Department of Neurosurgery, The First People's Hospital of Taicang, Taicang Affiliated Hospital of Soochow University, Soochow Medical College of Soochow University, Suzhou, Jiangsu Province, 215400, China; c Department of Neurosurgery, The First People's Hospital of Zhangjiagang, Jiangsu Province, 215600, China

**Keywords:** Post-stroke insomnia, Sedative-hypnotic, Treatment, Meta-analysis

## Abstract

**Background:**

：Post-stroke insomnia（PSI）is one of the common complications after stroke and it is a chronic problem and hampers the patient's recovery. Some treatments have been shown to be effective in treating post-stroke insomnia. However, it is not clear which treatment is more effective.

**Methods:**

In this study, we searched CNKI, PubMed, and Cochrane Library for appropriate keywords with a deadline of October 2022 to select 23 randomized controlled trials（RCTs）. The mean difference between different treatments was assessed and summarized as mean and 95 % confidence interval (CI), resulting in a Bayesian network meta-analysis.

**Results:**

By meta-analysis of Bayesian networks, we found acupuncture(MD, −2.49; 95 % CI, [-3.63, −1.31]) and herbal (MD, −2.79; 95 % CI, [-4.9, −0.69]) were significantly better than estazolam in terms of PSQI score change, and the difference was statistically significant. Dexzopiclone, electrics stimulation, rTMS and zopiclone were not statistically significant with other treatments.

**Conclusion:**

Herbal, zopiclone, and acupuncture were more effective in improving PSQI scores in patients with post-stroke insomnia, followed by rTMS and dexzopiclone. However, the effectiveness between herbal, zopiclone and acupuncture was not statistically significant. Acupuncture and herbal are promising for the treatment of PSI, and more research remains to be invested.

## 1 Introduction

Stroke is currently the second most common disease worldwide in terms of mortality and disability. Stroke is mainly classified as ischemic stroke and hemorrhagic stroke, and in 2010, the global incidence of these two major stroke types was about 11.6 million and 5.3 million. In 2016, the number of incident new strokes increased to 13.7 million [1]. Epidemiologically, the major risk factors for stroke include high blood ′pressure, smoking, diabetes, lack of exercise, and excessive alcohol consumption [2]. Stroke is a neurological disease, which is caused by different factors that lead to the formation of blood clots in the brain. The clots form and attach to the walls of the blood vessels, thus blocking the blood vessels leading to the accumulation of blood in the vessels, increasing the pressure in the walls of the vessels, followed by the rupture of the vessels and bleeding, which leads to a series of complications [3]. Common post-stroke complications include post-stroke epilepsy, post-stroke depression (PSD), post-stroke insomnia(PSI), anxiety disorders and post-stroke fatiguee [4,5]. The prevalence of insomnia after stroke has increased significantly over the years, with prevalence rates of 40.7 %, 42 %, and 47 % in the acute, subacute, and chronic phases of stroke, respectively. This indicates that more and more patients will be troubled by this problem, which will bring great psychological and physical suffering to the patients [6].

As one of the common complications after stroke, post-stroke insomnia is also highly disabling and dangerous. According to studies, at least 50 % of patients will develop post-stroke insomnia after stroke, and about 1/3 of them will develop insomnia after the first illness, while the rest of patients already have this symptom before the illness and are troubled by it [7]. In a study it was found that patients with post-stroke insomnia had a high probability of suicidal ideation. However, this problem is often overlooked in clinical practice, resulting in irreparable damage to the patient. This requires early prevention, early detection and early treatment of this type of insomnia in our daily clinical work [8]. A recent study also showed that post-stroke insomnia increases the risk of cognitive impairment, which can also hinder the subsequent recovery process and outcome of patients [9]. According to the research, he common causes of insomnia after stroke are environmental factors and insomnia caused by other post-stroke complications. The former is more commonly associated with light and noise effects, while the latter is often closely related to pain, depression, and Sleep-related breathing disturbances [10,11]. In addition the thalamus has been shown to be a key link in the regulation of wakefulness-sleep, and experiments have shown that thalamic cats can suffer from more severe and prolonged insomnia, which is caused by the loss of the ability to produce EEG sleep patterns after involvement of the anterior or dorsal nucleus discs of the thalamus [12]. Therefore, post-stroke damage to the thalamus or other brain tissues related to sleep may cause symptoms of insomnia. It has been suggested that post-stroke insomnia not only aggravates the depressed state of mind of patients after stroke onset, but also seriously affects the recovery process and efficiency of patients after stroke, or worse, persistent insomnia after stroke may in turn lead to stroke formation and recurrence of stroke [13,14].

Despite the favorable outcomes of thrombolysis and thrombolysis after acute ischemic stroke, patients still have varying degrees of post-stroke complications. As research on postsecondary insomnia continues, more and more treatment modalities are being applied in clinical work [15]. The main treatments for post-stroke insomnia currently is sedative-hypnotic drugs. Some benzodiazepines, such as estazolam and alprazolam, are often used clinically, and their mechanism of action is to positively modulate gamma-aminobutyric acid (GABA), the major inhibitory neurotransmitter in the mammalian nervous system, thereby enhancing the inhibitory effects of GABA. There are also non-benzodiazepine drugs that are often used in clinical practice, such as zopiclone, a representative of Z-drugs, whose mechanism of action is similar to that of benzodiazepines, but whose side effects are slightly less severe than those of benzodiazepines. However, this drug treatment often causes side effects, such as dependence, hangover effects, cognitive or memory impairment, and balance disorders [16,17]. In recent years, more and more new treatments have been used to treat post-stroke insomnia, such as repetitive transcranial magnetic stimulation(rTMS) and dexzopiclone [18]. In addition, a growing body of research shows that acupuncture and herbal therapies can also improve post-stroke insomnia. Acupuncture can regulate sleep by using needles to stimulate specific points on the body, and herbal therapies can reduce insomnia by regulating the neurotransmitter aminobutyric acid and inhibiting 5-hydroxytryptamine receptors through some sedative herbs [19,20].

Currently, there are many treatments for post-stroke insomnia, all of which are effective, but it is not clear which is the most effective. Although there have been several meta-analyses examining the efficacy of post-stroke insomnia, these studies only analyzed one of treatments and did not do a two-by-two comparison of the different treatments. Therefore, we conducted a network meta-analysis of different treatments for post-stroke insomnia to analyze which treatment is the most effective and to provide a theoretical reference for the clinical treatment of post-stroke insomnia patients.

## 2 Materials and methods

### 2.1 Study protocol

Before starting this study, we prepared a draft study protocol following the format of the Cochrane Collaboration. The meta-analysis has not yet been registered.

### 2.2 Literature search

In this article, the researchers screened respectively eligible studies from CNKI, PubMed, and the Cochrane Library for various treatments for post-stroke insomnia. Dates searched are July 2000 to December 2022. The keywords searched were “post-stroke insomnia” AND (“acupuncture” OR “herbal” OR “estazolam” OR “zopiclone” OR “dexzopiclone” OR “alprazolam” OR “repetitive transcranial magnetic stimulation” OR “rTMS” OR “electrical stimulation”) AND (“randomized controlled trail” OR “RCT” OR “random” OR “controlled”).

### 2.3 Inclusion and exclusion criteria

For a comprehensive and objective analysis we included articles with the following criteria: (1) A randomized controlled study for the treatment of post-stroke insomnia. (2) Each included article had to include the indicator of the change in Pittsburgh Sleep Quality Index(PSQI). (3) Articles that meet the criteria must include at least one form of treatment, including acupuncture, alprazolam, dexzopiclone, electrics stimulation, herbal, rTMS, estazolam and zopiclone. (4) Patients participating in the study must meet the diagnostic criteria for post-stroke insomnia.

### 2.4 Quality assessment and data extraction

For all eligible articles included, we used the Cochrane Collaboration Risk Bias Assessment Tool to perform a holistic assessment [21]. We independently extracted data from the included eligible articles by two reviewers separately, including first author, year of publication, sample size of included patients, patient age and gender ratio. In addition, if there is a disagreement between two reviewers in the process of data extraction and quality assessment, a third party reviewer will discuss and reach a conclusion.

### 2.5 Statistical analysis

For the extracted data, we used R4.0.3 software to construct a constructed Bayesian network model and performed conventional paired meta-analysis and network analysis. In addition, we used Review Manager 5.4.1 to perform bias analysis and sensitivity analysis on the extracted data. We first verified the heterogeneity of the constructed models, which was assessed using the chi-square Q test and the I2 statistic. If I2 > 50 % showed significant heterogeneity, a random effects model was used. Conversely, a fixed effects model was used. We then verified the consistency in the network model by comparing the presence of direct, indirect, and pooled evidence at each node. The statistical differences between different evidence were assessed using node splitting to determine the consistency of indirect evidence with direct evidence. Finally we showed the results of the comparison between different treatments by constructing forest plots, league tables and ranking plots [22,23].

## 3 Results

### 3.1 Study characteristics

**Fig. 1:**
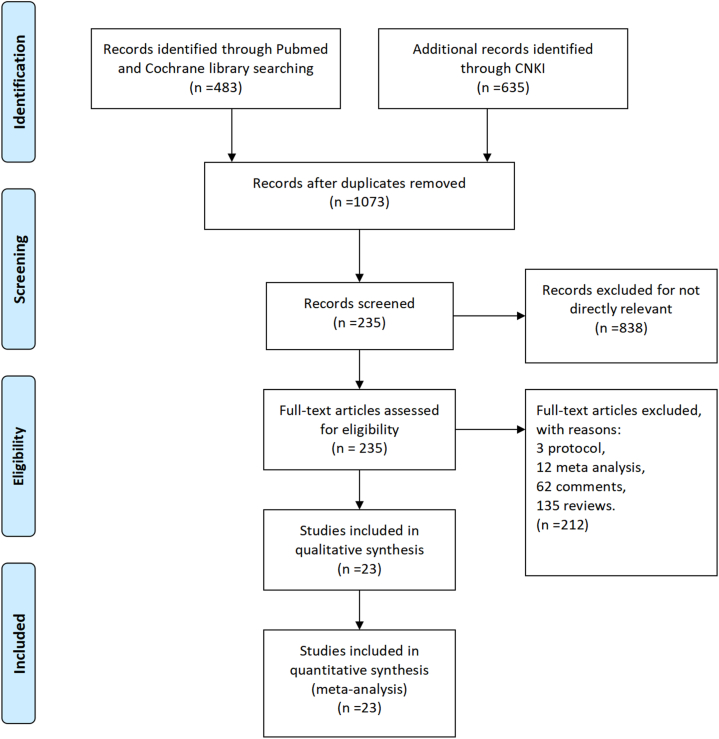
flow diagram for study identification.

Based on the above method, we retrieved 483 articles in PubMed and Cochran Library and 635 articles in CNKI. By comparison, we eliminated the duplicate articles and removed 838 articles by preliminary reading of the abstracts. And then again by reading the full text, 3 protocols, 12 meta-analyses, 62 comments and 135 reviews were removed. In the end, only 23 articles with characteristic information were included in this paper, as shown in Fig. 1, which includes 8 treatment modalities, including acupuncture, herbal therapies, estazolam, zopiclone, dexzopiclone, alprazolam, electrical stimulation and rTMS. The characteristics of the final included studies are shown in Table 1. In this study, the 23 randomized controlled trials included involve a total of 1593 patients [[24], [25], [26], [27], [28], [29], [30], [31], [32], [33], [34], [35], [36], [37], [38], [39], [40], [41], [42], [43], [44], [45], [46]]. Among them, 516 patients were treated with acupuncture, 563 patients with estazolam, 192 patients with herbal therapies, and 40 patients with electrical stimulation, 28 patients received repetitive transcranial magnetic stimulation, 30 patients received zopiclone, 75 patients received dexzopiclone, and 149 patients received alprazolam. The patients included in this study were mostly male and more than 50 years old.

**Table 1:** Characteristics of the included studies and outcome events.

Study	Countries	Publications	Treatment group, (No. of participants)	Scores of PSQI (Mean ± SD)	Female (%)	Mean age ±SD (year)	Treatment cycles
Li (2007)	China	Chinese Journal of Rehabilitation Theory and Practice	Acu (32) vs.Est (32)	Acu 21.18 ± 4.21 Est 22.49 ± 4.17	Acu 44％ Est 47％	Acu 69.8 ± 7.1 Est 67.3 ± 8.3	4 weeks
Sun and Xia (2011)	China	Shanghai Journal of Acupuncture and Moxibustion	Acu (30) Vs.Est (30)	Acu 13.31 ± 2.41 Est 12.09 ± 3.71	Acu 53 % Est 50 %	Acu 40 ± 15 Est 40 ± 15	3 weeks
Xu ang Li (2012)	China	Nei Mongol Journal of Traditional Chinese Medicine	Her (31) Vs.Alp (31)	Her 13.65 ± 2.51 Alp 14.58 ± 2.89	Her 55 % Alp 61 %	Her 72.2 ± 4.8 Alp 70.2 ± 3.9	4 weeks
Ye etc (2013)	China	Chinese Journal of New Clinical Medicine	Acu (43) Vs.Alp (42)	Acu 14.88 ± 3.27 Alp 15.50 ± 3.50	Acu 47 % Alp 52 %	Acu 62.8 ± 7.2 Alp 67.3 ± 8.3	4 weeks
Tang etc (2014)	China	Chinese Acupuncture & Moxibustion	Lfes (40) Vs.Est (40)	Lfes 13.30 ± 1.38 Est 13.25 ± 1.25	Lfes 45 % Est 48 %	Lfes 63 ± 11 Est 65 ± 10	4 weeks
Luo and Wu (2015)	China	Guangdong Medical Journal	Dex (45) Vs.Alp (45)	Dex 15.15 ± 2.21 Alp 15.01 ± 2.53	Dex 49 % Alp 47 %	Dex 59.7 ± 10.3 Alp 61.5 ± 8.5	4 weeks
Tang and Zhang (2015)	China	Chinese Journal of Integrative Medicine on Cardio-/Cerebrovascular Disease	Acu (34) Vs.Est (31)	Acu 13.62 ± 4.82 Est 12.98 ± 4.21	Acu 47 % Est 45 %	Acu 58.25 ± 9.31 Est 59.68 ± 8.73	4 weeks
Ma etc (2016)	China	China Medical Herald	Acu (40) Vs.Est (40)	Acu 14.85 ± 4.52 Est 15.45 ± 4.70	Acu 40 % Est 50 %	Acu 61.88 ± 5.16 Est 63.70 ± 4.94	4 weeks
Gao (2016)	China	China Journal of Traditional Chinese Medicine and Pharmacy	Her (20) Vs.Est (20)	Her 17.50 ± 2.95 Est 16.75 ± 2.86	Her 55 % Est 60 %	Her 61.50 ± 7.25 Est 59.90 ± 8.72	2 weeks
Zhang (2016)	China	China Journal of Traditional Chinese Medicine and Pharmacy	Acu (50) Vs.Est (53)	Acu 17.79 ± 1.31 Est 17.90 ± 1.29	Acu 48 % Est 53 %	Acu 62.30 ± 8.02 Est 63.92 ± 7.44	4 weeks
Li (2018)	China	Journal of Basic Chinese Medicine	Acu (23) Vs.Est (33)	Acu 14.24 ± 2.40 Est 14.07 ± 1.69	Acu 43 % Est 52 %	Acu 55.52 ± 10.01 Est 57.19 ± 9.92	2 weeks
Yang (2018)	China	Modern Journal of Integrated Traditional Chinese and Western Medicine	Acu (30) Vs.Est (30)	Acu 17.21 ± 3.72 Est 16.84 ± 4.07	Acu 40 % Est 27 %	Acu 70.09 ± 12.90 Est 67.50 ± 11.57	2 weeks
Wei (2019)	China	World Journal of Sleep Medicine	Her (31) Vs.Alp (31)	Her 17.86 ± 1.32 Alp 18.02 ± 1.27	N/A	N/A	12 weeks
Le (2020)	China	Jiangxi Medical Journal	Acu (30) Vs.Dex (30)	Acu 14.24 ± 2.40 Dex 14.07 ± 1.69	Acu 33 % Dex 30 %	Acu 55.52 ± 10.01 Dex 57.19 ± 9.92	4 weeks
LI (2020)	China	World Chinese Medicine	Acu (50) Vs.Est (50)	Acu 14.15 ± 4.13 Est 13.78 ± 3.95	Acu 40 % Est 44 %	Acu 62.35 ± 11.82 Est 61.90 ± 10.23	4 weeks
Zhang (2020)	China	Lishizhen Medicine and Materia Medica Research	Acu (29) Vs.rTMS (28)	Acu 12.6 ± 2.7 rTMS 12.5 ± 3.4	Acu 86 % rTMS 82 %	Acu 49.8 ± 9.2 rTMS 51.3 ± 8.7	4 weeks
Zhang (2020)	China	China Journal of Traditional Chinese Medicine and Pharmacy	Acu (15) Vs.Est (17)	Acu 14.53 ± 2.07 Est 14.76 ± 1.99	Acu 47 % Est 41 %	Acu 60.07 ± 9.37 Est 63.76 ± 6.92	1 weeks
He (2020)	China	Practical Journal of Cardiac Cerebral Pneumal and Vascular	Acu (30) Vs.Est (30)	Acu 14.9 ± 2.4 Est 14.6 ± 2.6	N/A	N/A	2 weeks
Dai etc (2020)	Switzerland	Frontiers in Neurology	Her (30) Vs.Zop (30)	Her 13.20 ± 2.75 Zop 14.50 ± 2.87	Her 43 % Zop 50 %	Her 59.87 ± 8.32 Zop 60.47 ± 9.21	4 weeks
Gao (2021)	China	World Journal of Sleep Medicine	Her (40) Vs.Est (40)	Her 15.36 ± 3.98 Est 15.54 ± 5.01	N/A	N/A	2 weeks
Yu (2021)	China	Chinese General Practice	Acu (30) Vs.Est (30)	Acu 15.78 ± 2.74 Est 15.62 ± 1.53	Acu 60 % Est 53 %	Acu 65.0 ± 10.5 Est 65.0 ± 10.1	4 weeks
Zhao (2021)	China	Chinese Journal of Integrative Medicine on Cardio-/Cerebrovascular Disease	Her (40) Vs.Est (40)	Her 15.16 ± 2.03 Est 14.87 ± 1.95	Her 53 % Est 55 %	Her 52.7 ± 6.1 Est 53.5 ± 5.9	4 weeks
Zhang (2022)	China	China Journal of Traditional Chinese Medicine and Pharmacy	Acu (50) Vs.Est (47)	Acu 14.32 ± 2.89 Est 13.85 ± 2.54	N/A	Acu 67.38 ± 2.98 Est 67.62 ± 3.60	N/A

Acu: acupuncture; Es: electrical stimulation;rTMS: repetitive transcranial magnetic stimulation; Est: estazolam; Her: herbal; Zop: zopiclone; Dex: dexzopiclone; Alp: alprazolam.; N/A: not application.

### 3.2 Quality assessments of the selected literature

**Fig. 2:**
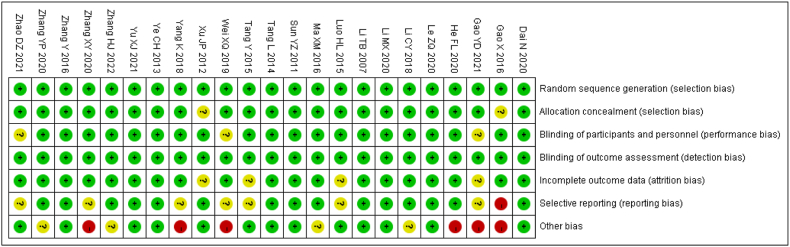
Risk of bias summary:review authors' judgments about each risk of bias item for each included study.

We performed a more detailed bias analysis of the 23 articles included. The factors that may have influenced the bias were mainly the size of the included population, the sex ratio and the mean age ratio, which are indicated in the “Other bias” item, such as Gao (2016), Li (2018) and Zhang (2020) included a sample size of only 40, 56 and 32, which is small compared to other included articles. The sex ratio of Zhang (2022) is unknown, The mean age of Wei (2019) is unknown, and the gender and mean age proportions of both He (2020) and Gao (2021) are unknown. In addition, in Dai (2020), there is some selection bias, and the authors' allocation of the sample is not fully randomized, which could also bias our results (Fig. 2).

### 3.3 Network meta-analysis

**Fig. 3:**
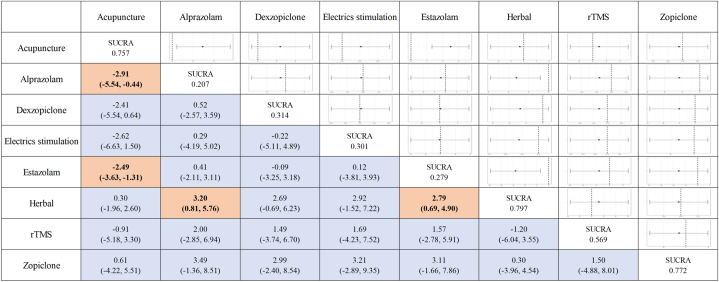
league table for outcome of the change in PSQI score using the random effect model.

We analyzed the efficacy of different treatments for post-stroke insomnia by network meta-analysis, including acupuncture, alprazolam, dexzopiclone, electrics stimulation, herbal, rTMS, estazolam and zopiclone. we selected the index of the change in PSQI score to evaluate the effectiveness of different treatments. In this study, patients receiving acupuncture (MD, −2.45; 95 % CI, [−3.63, −1.31]), and herbal (MD, −2.79; 95 % CI, [−4.9, −0.69]) were significantly superior compared to estazolam. The remaining treatments were not statistically significant compared to estazolam (Fig. 3).

### 3.4 Rank probability

**Fig. 4:**
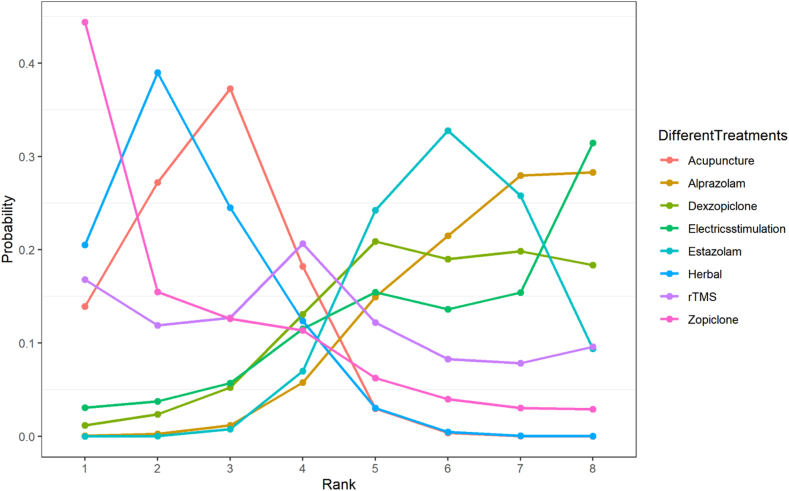
Line graph for probability rank of the change in PSQI socre.

To show the probability ranking of each treatment strategy corresponding to the indicator, we drew a line graph based on the evidence from the network (Fig. 4). The line graph of the probability ranking for the indicator was analyzed together with the SUCRA values in Fig. 3. In our study, herbal (SUCRA, 0.797) showed the best improvement in PSQI scores, while zopiclone (SUCRA, 0.772) showed the second best improvement and acupuncture (SUCRA, 0.757) showed the third best improvement. On the contrary, alprazolam (SUCRA, 0.207) was the last. Of interest, although zopiclone had the highest probability of ranking first in the probability ranking, the sucra value was lower than herbal therapies and therefore ranked second in the effectiveness of different treatments for treating post-stroke insomnia. Of course, there is no statistical significant difference between zopiclone and herbal therapies in terms of efficacy in treating post-stroke insomnia.

### 3.5 Heterogeneity examination

**Fig. 5:**
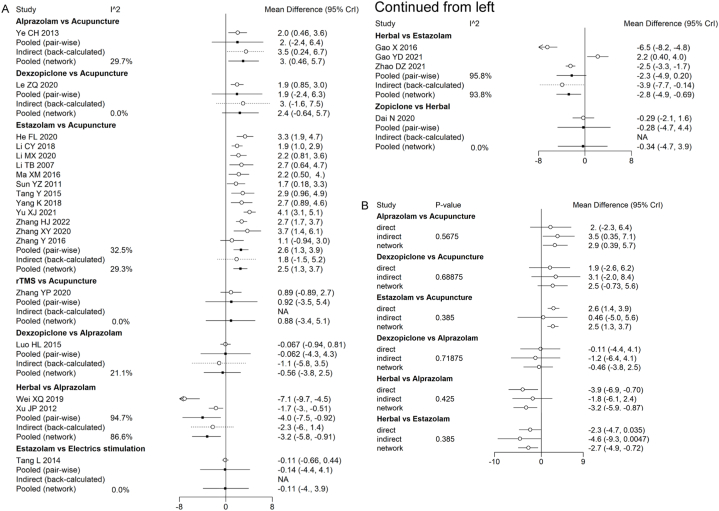
Forest plotes for the heterogeneity and consistency of the chang in PSQI socre.(A)Heterogeneity.(B)Consistency.

Before constructing the final network model, we conducted a heterogeneity analysis of direct, indirect, and pooled evidence for the indicator. Since the overall I2 for this indicator of the change in PSQI score was >50 %, we used a random-effects model. The I2 values for each evidence from different studies are presented in Fig. 5A.

### 3.6 Consistency and similarity examinations

Direct, indirect, and pooling evidences are available in the network for the indicator. Therefore, we used the node-splitting model to test the differences between direct and indirect comparisons and perform consistency tests. We found no significant inconsistencies in the direct and indirect evidence, the results of which were shown in Fig. 5B, with all P values > 0.05. This showed that the consistency model we constructed was reliable. We tested sensitivity by bias analysis and quality control of the included articles. According to the previous results, no significant differences were shown between data.

## 4 Discussion

Post-stroke insomnia (PSI) is a common post-stroke complication that severely affects the quality of life of patients. There is evidence that insomnia may also be a high risk factor for stroke, this will cause the patient to have another stroke. It has been reported in the literature that strokes in the dorsal or tegmental, parthalamic or lateral or subcortical regions of the brainstem are more likely to cause insomnia. Although the exact mechanism of stroke-induced insomnia is not well understood, prevention and treatment of PSI remains a concern for frontline clinicians [47]. Currently, sedative-hypnotic drugs are often used in clinical treatment of post-stroke insomnia, such as estazolam, zopiclone, dexzopiclone. As research into post-stroke insomnia has progressed in recent years, more and more treatment modalities have been shown to be effective, including acupuncture, herbal therapies, electrical stimulation and rTMS [48,49]. However, it is not known which treatment is more effective. In this study, we performed a Bayesian meta-analysis of the 23 articles included to compare the effectiveness of treatments. In our study, we found that the herbal, zopiclone and acupuncture were more effective in treatments of post-stroke insomnia. This will provide some theoretical evidence for future clinical treatment.

Through an extensive search of the relevant literature, we finally chose the pittsburgh sleep quality index (PSQI) score change as an outcome indicator to measure the effectiveness of different treatments for post-stroke insomnia. PSQI is the most commonly used clinical scale to measure sleep quality, and it is not only applicable to people with sleep disorders caused by various causes or diseases, but also to the normal population. Its main content is divided into seven parts, including subjective sleep quality, sleep latency, sleep duration, habitual sleep efficiency, sleep disorders, sleep medication use, and daytime dysfunction. The whole scoring process takes about 5–10 min, which is faster and easier compared to other scores such as Insomnia Severity Index(ISI). In addition, the total score of PSQI ranges from 0 to 21, the higher the score, the worse the sleep quality [50,51]. We only study here the amount of change in the overall PSQI with the aim of assessing which treatment is more efficacious. In the included articles.

In this network meta-analysis, we found that all the included treatment modalities improved the PSQI scores of post-stroke insomnia patients. Among them, herbal (SUCRA, 0.797) ranked first, zopiclone (SUCRA, 0.772) ranked second, and acupuncture (SUCRA, 0.757) ranked third. As estazolam, which is often used in clinical treatment, ranked only 7th, both acupuncture (MD, −2.49; 95 % CI, [−3.63, −1.31]) and herbal (MD, −2.79; 95 % CI, [−4.9, −0.69]) were superior to estazolam, and the difference was statistically significant. Estazolam has been shown to treat post-stroke insomnia by binding to gamma-aminobutyric acid (GABA) receptors to enhance the inhibition of synaptic transmission mediated by GABA. In addition, estazolam has minimal hepatotoxicity compared to other benzodiazepines. Furthermore, studies have shown that older adults are more sensitive to estazolam than younger adults [52]. Therefore, estazolam is currently used as the most common treatment for post-stroke insomnia. Since we included RCTs mostly comparing estazolam with other treatments and did not have placebo group patients, we chose to compare the efficacy of different treatments with estazolam in our network meta-analysis. With continued research into herbal therapies and acupuncture, they have been found to be effective in relieving a variety of mental illnesses such as insomnia, depression and cognitive disorders. For example, Xiao Chai Hu decoction may alleviate patients' anxiety symptoms by decreasing the levels of factors related to brain neurological function such as S100B, MBP, NSE and IGF-1 [53]. Acupuncture can adjust the patient's sleep by stimulating different acupoints. However, the specific mechanism of herbal and acupuncture for post-stroke insomnia is not well understood and further research is needed. Our network meta-analysis likewise found zopiclone to be equally effective in the treatment of post-stroke insomnia. This may be due to the small number of RCTs we included, only 1, and the small sample size. Therefore further studies are needed for the effectiveness of zopiclone. It is worth noting that although herbal, zopiclone, and acupuncture were ranked according to the SUCRA values, there was actually no statistical significance between these 3 treatments. This is because the ranking of the effectiveness of different treatments based on SUCRA values is an estimate of likelihood [54]. In addition, benzodiazepines, including estazolam and alprazolam, often have short-term adverse events, such as dizziness and weakness [55]. In contrast, acupuncture and herbal therapies have fewer adverse events. Because datas on adverse events were not available for the RCTs we included, further research is needed on the safety of different treatments for post-stroke insomnia. Combined with the results of our network meta-analysis, herbal, zopiclone, and acupuncture were more effective in improving PSQI scores in patients with post-stroke insomnia, and herbal and acupuncture were significantly more effective than estazolam. Of course, there are many confounding factors that affect insomnia in daily life, including the participants' age, stroke severity, comorbidities, etc. Therefore further subgroup analyses are needed in the future to evaluate the effectiveness of different treatments for post-stroke insomnia.

However, there are some limitations of this study. Firstly, the sample size of patients included in this paper for various treatment modalities is small, and therefore, the results of this paper can only reflect the impact of small studies. Secondly, the PSQI scores were mostly self-rated by patients themselves, and the results may also be subjective to patients, which may also lead to biased results. Thirdly, our data and conclusions are based on statistical analysis, and the clinical validity of this method is not currently supported by sufficient data in the literature. The firth is this study only provides a limited comparative analysis of the effectiveness of acupuncture, herbal medicine, and valium medications, and no analysis of their safety, adverse effects, or other aspects was performed. Fifthly, only the Pittsburgh Sleep Index Scale was used as the primary outcome in this article, The effect of different treatment modalities on post-stroke insomnia cannot be fully demonstrated. The sixth is that the number of RCTs for zopiclone for post-stroke insomnia was small, with only 1. This would create some bias in the study results.

According to the network meta-analysis, herbal, zopiclone, and acupuncture were more effective in improving PSQI scores in patients with post-stroke insomnia, followed by rTMS and dexzopiclone. Among them, herbal and acupuncture were significantly better than estazolam in improving patients' PSQI scores, and the effectiveness between herbal, zopiclone and acupuncture was not statistically significant. Further studies are needed on the safety of different treatments for post-stroke insomnia.

## Author contribution statement

All authors listed have significantly contributed to the development and the writing of this article.

## Data availability statement

All data generated or analyzed during this study are included in this published article and its supplementary information files.

## Ethics approval and consent to participate

Not applicable.

## Declaration of competing interest

The authors declare that there is no conflict of interest in our study.

## Abbreviation

RCT: randomized controlled trial
MD: mean difference
SUCRA: surface under the cumulative ranking curve
CI: confidence interval
