# Supplementary material for: Comparative effectiveness of different treatments for post-stroke insomnia: A network meta-analysis
Source: Heliyon. 2023 Oct 31;9(11):e21801. doi: 10.1016/j.heliyon.2023.e21801 (PMC10658229; doi:10.1016/j.heliyon.2023.e21801)
Supplement: Multimedia component 1 [file mmc1.docx]

**Comparative Effectiveness of Different Treatments for Post-Stroke Insomnia: A Network Meta-Analysis**

**Zhaoming Song^1,#^, Ying Chen^2^,#, Jian Li^3^, Zhouqin Chen^1^, Xiaojun Lu^2,*^, Zhong Wang ^1,*^**

***^1^ Department of Neurosurgery & Brain and Nerve Research Laboratory, The First Affiliated Hospital of Soochow University, Suzhou, Jiangsu Province, 215006, China***

***^2^ Department of Neurosurgery, The First People's Hospital of Taicang City, Taicang Affiliated Hospital of Soochow University, Suzhou, Jiangsu Province, 215400, China***

***^3^ Department of Neurosurgery, The First People's Hospital of Zhangjiagang City, Jiangsu Province, 215600, China***

**Supplement I**


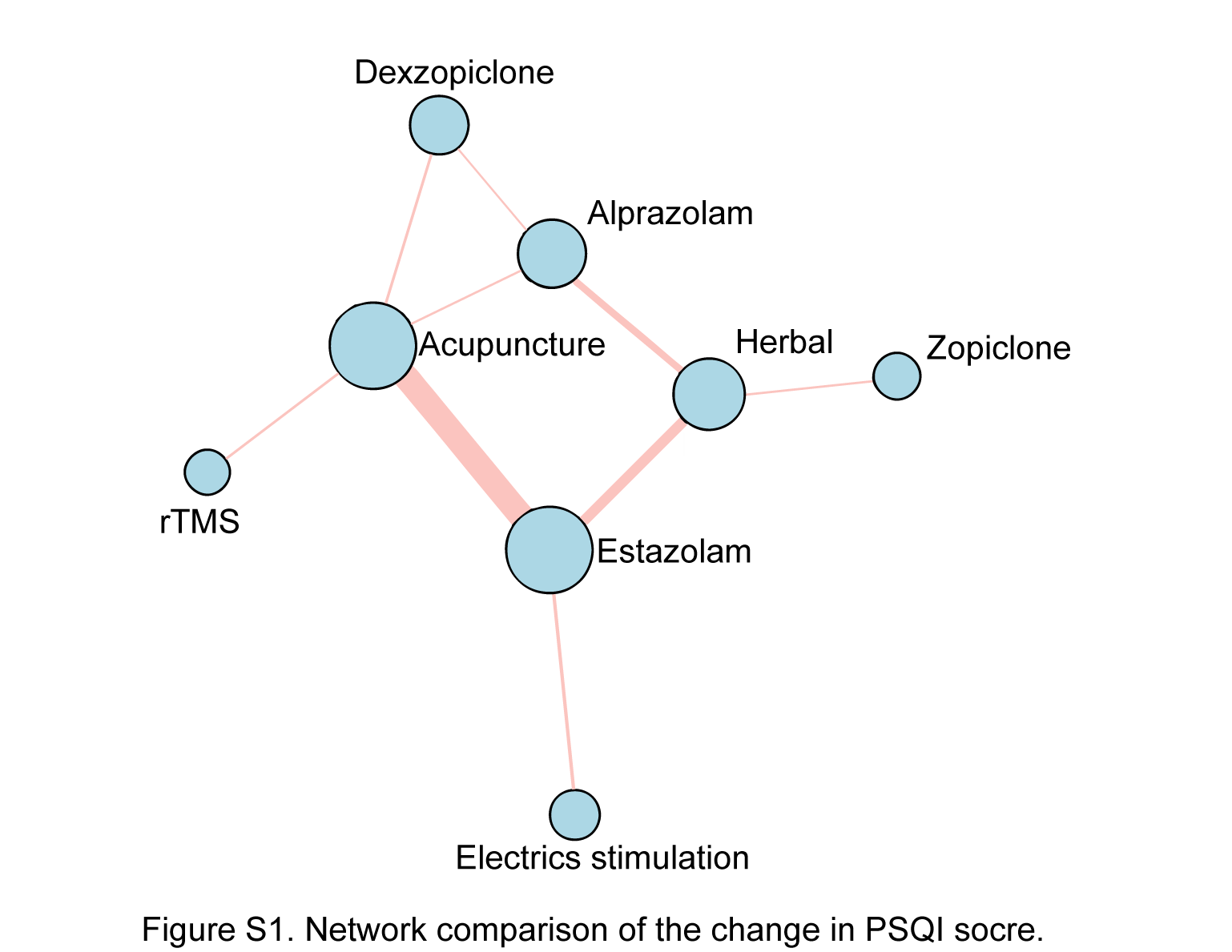
Figure S1. Network comparison of the change in PSQI socre.
